# Alliance and Treatment Outcome in Family-Involved Treatment for Youth Problems: A Three-Level Meta-analysis

**DOI:** 10.1007/s10567-017-0249-y

**Published:** 2017-12-07

**Authors:** M. J. Welmers-van de Poll, J. J. Roest, T. van der Stouwe, A. L. van den Akker, G. J. J. M. Stams, V. Escudero, G. J. Overbeek, J. J. W. de Swart

**Affiliations:** 1 Research Centre Youth Care, Windesheim University of Applied Sciences, Postbus 10090, 8000 GB Zwolle, The Netherlands; 2 Youth Expert Centre, Leiden University of Applied Sciences, Zernikedreef 11, Postbus 382, 2300 AJ Leiden, The Netherlands; 3 Child and Youth Care Sciences, University of Amsterdam, Nieuwe Achtergracht 127, 1018 WS Amsterdam, The Netherlands; 4 Department of Psychology, University of A Coruña, Elviña, 15071 A Coruña, Spain; 5 Research Group Social Work, Saxion University of Applied Sciences, Postbus 70.000, 7500 KB Enschede, The Netherlands

**Keywords:** Multilevel meta-analysis, Family-based treatment, Therapeutic alliance, Working alliance, Treatment outcomes

## Abstract

Alliance has been shown to predict treatment outcome in family-involved treatment for youth problems in several studies. However, meta-analytic research on alliance in family-involved treatment is scarce, and to date, no meta-analytic study on the alliance–outcome association in this field has paid attention to moderating variables. We included 28 studies reporting on the alliance–outcome association in 21 independent study samples of families receiving family-involved treatment for youth problems (*N* = 2126 families, *M* age youth ranging from 10.6 to 16.1). We performed three multilevel meta-analyses of the associations between three types of alliance processes and treatment outcome, and of several moderator variables. The quality of the alliance was significantly associated with treatment outcome (*r* = .183, *p* < .001). Correlations were significantly stronger when alliance scores of different measurement moments were averaged or added, when families were help-seeking rather than receiving mandated care and when studies included younger children. The correlation between alliance improvement and treatment outcome just failed to reached significance (*r* = .281, *p* = .067), and no significant correlation was found between split alliances and treatment outcome (*r* = .106, *p* = .343). However, the number of included studies reporting on alliance change scores or split alliances was small. Our findings demonstrate that alliance plays a small but significant role in the effectiveness of family-involved treatment. Future research should focus on investigating the more complex systemic aspects of alliance to gain fuller understanding of the dynamic role of alliance in working with families.

## Introduction

In the treatment of mental health or behavior problems of children and adolescents, involving the family can be an important part of the intervention. Given the influence of family functioning on child and adolescent development (Rutter 2002), treatment to target problematic family functioning and to enhance protective family factors can be vital in reducing youth psychopathology. Indeed, results of several randomized controlled trials support the effectiveness of family-based treatment models for youth problems, such as attachment-based family therapy (ABFT; Diamond et al. 2010), multidimensional family therapy (MDFT; Henderson et al. 2010; Rigter et al. 2013), functional family therapy (FFT; Hartnett et al. 2016; Sexton and Turner 2011) and family-based therapy (FBT; Couturier et al. 2013; Lock et al. 2010). Moreover, in comparative meta-analytic reviews on the effectiveness of treatment for youth delinquency (Latimer 2001), adolescent substance abuse (Tanner-Smith et al. 2013) and anorexia nervosa (Lock et al. 2010), family treatment models have been shown to be more effective than interventions for youth only.

Over the past years, delivery of family-based interventions for youth has become more integrative and flexible, and interventions that combine individual therapy, family treatment and sometimes medication have become increasingly popular (Diamond and Josephson 2005). An example of such an integrative intervention is family-based cognitive behavioral therapy (FB CBT), which has shown to be efficacious for treatment of pediatric obsessive–compulsive disorder (O’Leary et al. 2009; Storch et al. 2007) and anxiety disorders (Ginsburg and Schlossberg 2002; Kendall et al. 2008).

In order to gain better understanding of the effectiveness of family-involved interventions, it is important to know what components or conditions of treatment cause positive outcomes. Previous research has shown that the alliance between therapists and clients is a significant predictor of treatment outcome in individual youth psychotherapy as well as family therapy (Friedlander et al. 2011; McLeod 2011; Shirk et al. 2011).

Most research on alliance is based on Bordin’s (1979) definition of the alliance which he developed for the individual therapy context, also referred to as therapeutic or working alliance. Bordin argues that the professional relationship between a therapist and client consists of three components: (a) an emotional bond between therapist and client based on mutual trust and sympathy, (b) agreement on which problems and goals are the central issue in therapy and (c) agreement on tasks that need to be performed by therapist and client in order to achieve central goals.

The process of building and maintaining an emotional bond and agreement on tasks and goals raises several complexities in working with families. In family-involved treatment, the therapist simultaneously develops multiple alliances with family members who are in treatment together, but who differ in their characters, needs and treatment expectations (Kindsvatter and Lara 2012; Rait 2000). For instance, in a study on alliance and treatment outcome in home-based family therapy by Johnson et al. (2002) the correlation between alliance and outcome was stronger for fathers than for mothers. For fathers, the agreement with the therapist about treatment goals was more predictive of treatment outcome than the agreement on tasks and the emotional bond, whereas for mothers agreement on tasks was relatively more predictive of treatment effectiveness. In addition, research showed that treatment effectiveness can be reduced when the therapist develops a stronger alliance with one family member than with the other: These unbalanced or so-called split alliances increase the risk of treatment drop out (Flicker et al. 2008; Robbins et al. 2003).

Another complicating aspect of building and maintaining alliances in family-involved treatment is that each person’s alliance with the therapist is observed and influenced by the other participating family members (Friedlander et al. 2006; Kindsvatter and Lara 2012). These observations might cause feelings of unsafety or anxiety, since what is said during a session can have repercussions outside therapy sessions. For example, a teenage son who tells the therapist about a relapse in drug abuse with his parents present might be worried about getting punished at home for this relapse. Thus, the therapist needs to provide guidelines or discuss basic rules of safety and confidentiality in order to gain confidence and trust from all participating family members (Friedlander et al. 2006).

A third aspect of alliance specific to family-involved treatment is that treatment outcome is not only affected by multiple individual alliances between therapist and family members, but also by the alliance with family as a whole (Escudero et al. 2008; Friedlander et al. 2008; Kindsvatter and Lara 2012). When family members perceive themselves as a group collaborating to improve family functioning and achieve other therapeutic goals, treatment is more likely to be effective. Therefore, family therapists must leverage different views on problems and solutions within the family and try to bring about a shared sense of common family goals by for example emphasizing shared values and experiences (Escudero et al. 2008; Friedlander et al. 2006; Rait 2000).

Perhaps because of these complexities in alliance processes specific to family-involved treatment, research on alliance in this field emerged later and received far less attention than research on alliance in individual psychotherapy. In the 1980s, Pinsof and Catherall (1986) applied Bordin’s definition of alliance to three interpersonal levels by measuring bonds, tasks and goals for three relationships: self with therapist, other with therapist and group with therapist. This approach was elaborated on by Pinsof (1994) when he added the *within*-*family alliance*, namely the extent to which family members collaborate on goals and tasks and experience an emotional bond with each other during therapy. Symonds and Horvath (2004) defined this concept as *allegiance.* Friedlander et al. (2006) elaborated on Bordin’s definition of alliance as well as family therapy-specific alliance processes, such as allegiance, by distinguishing four domains of alliance in family therapy: (a) emotional connection to the therapist, (b) engagement in the therapy, (c) shared sense of purpose within the family (similar to Pinsof’s *within*-*family alliance*) and (d) safety within the therapeutic system. The two latter domains are said to be unique to conjoint family therapy.

To date, only one meta-analytic review on the association between alliance and outcome in family-involved treatment has been published (Friedlander et al. 2011). This study investigated the alliance–outcome correlation in 16 family therapy studies and 8 couple therapy studies. The result of the analysis was an average weighted effect size of *r* = .24 for the family therapy studies, demonstrating that higher levels of alliance are associated with more positive treatment outcome. This overall effect size is comparable to the effect size in meta-analyses on alliance and outcome in individual adult and youth psychotherapy (Horvath et al. 2011; Shirk et al. 2011).

Although Friedlander et al.’s (2011) meta-analysis provides a valuable test of the association between alliance and outcome in family therapy, the study also underlines the importance of further meta-analytical research on alliance in family-involved treatment for two reasons. First, the study included only 16 family therapy studies published until 2008. Since then, scientific attention for alliance processes in family-involved treatment research has burgeoned, resulting in an increase in studies on the subject. Second, the study reported significant variability in the correlation between alliance and outcome. This is not surprising, because the studies that were included in the meta-analysis showed a large heterogeneity with regard to alliance measures and other methodological aspects. This variety within and between studies was dealt with by collapsing several alliance measures (e.g., multiple types of alliance, informants, measurement instruments and measurement moments) into one effect size per study. No distinction was made between different types of alliance processes, and no moderator analyses were conducted. Therefore, the reported variability between studies remained unexplained.

### Different Types of Alliance Processes in Family-Involved Treatment

In research on the association between alliance and outcome in family-involved treatment, different types of alliance processes can be distinguished. A first type of alliance is the more traditional fixed moment measure of the level of alliance. Alliance can be measured at the start, middle or end of therapy, or at multiple moments, emphasizing that alliance is an ongoing process rather than a fixed state concept (Horvath 2006; Karver and Carporino 2010). In addition, some studies use alliance change scores to investigate whether the improvement of alliance during the therapy process influences treatment outcome (e.g., Bachler et al. 2016; Keeley et al. 2011). The relevance of this second type of alliance is illustrated by a study on alliance in adolescent psychotherapy, demonstrating that alliance change scores explain more variance in treatment outcome compared to single-moment measures or an average of multiple single-moment measures (Owen et al. 2016).

A third type of alliance refers to so-called split or unbalanced alliances and addresses the systemic aspect of alliance in family-involved treatment. Multiple family members form alliances with the therapist, which might differ in strength. When one family member has a better alliance with the therapist than other family members (i.e., alliances with the therapist are unbalanced between family members), this is generally referred to as a “split alliance.” Some studies have investigated whether these split alliances affect treatment outcome by subtracting family members’ single alliance scores and correlating these discrepancy scores with treatment outcome. When discrepancy scores are investigated, a negative correlation with treatment outcome is expected (i.e., higher levels of unbalance lead to less favorable treatment outcomes) instead of a positive correlation, as is the general hypothesis in research on the level of individual or family alliance and outcome.

### Moderators of the Alliance: Outcome Association

The association between alliance and outcome can be moderated by several factors. Several methodological aspects of studies might have a moderating effect, as has been reported in meta-analyses on alliance and outcome in youth and adult psychotherapy (Horvath et al. 2011; McLeod 2011; Shirk and Karver 2003; Shirk et al. 2011). First, it is important to investigate whether study quality moderates the alliance–outcome association: When higher quality studies indicate a stronger effect, this might be an indication of the robustness of the association. Second, timing of alliance measurement can be an important moderator. Alliance might be a predictor of outcome early in treatment, underlining the importance of alliance as a facilitator of successful therapy. On the other hand, meta-analyses in youth psychotherapy (McLeod 2011; Shirk and Karver 2003) and adult psychotherapy (Horvath et al. 2011) have indicated that alliance might be more predictive of outcome when assessed in a later stage of treatment, as it may need some time to build.

It might furthermore be of influence whose perspective on alliance as well as on outcome is measured (parent, youth, therapist or observer). Especially in family-involved treatment, with multiple family members involved, it is important to know what perspective is most predictive of successful treatment. Meta-analyses on alliance in youth psychotherapy either suggest that the parents’ or the therapists’ perspective on the alliance is most predictive of outcome (McLeod 2011; Shirk and Karver 2003) and that children’s reports on the alliance show very little variability (Shirk and Karver 2003). In addition, alliance seems to be most predictive of therapeutic outcome as perceived by either the parent (McLeod 2011) or the therapist (Shirk and Karver 2003) when compared to youth or observer reported outcome.

A methodological feature specific to studies on alliance in family-involved treatment is whether the alliance is measured at an individual (e.g., parent–therapist, youth–therapist) or family level (the alliance between the therapist and the family as a whole) using instruments specifically designed for family interventions. These instruments not only investigate individual alliances between family members and therapist, but additionally address the within-group or group-with-therapist aspects of alliance typical of family interventions. The moderating effect of type of alliance in family therapy is illustrated in a study by Escudero et al. (2008), in which the within-family alliance was correlated more strongly with outcome than the individual alliances. However, not all studies on alliance in family-involved treatment use instruments designed to measure family aspects of the alliance as well as individual alliances. As pointed out by McLeod (2011), the correlation between alliance and outcome in family-involved treatment might be stronger when the alliance measure is designed to investigate alliance processes typical of working with multiple family members.

Aside from methodological features of studies, several treatment aspects could moderate the effect of alliance on outcome. First, treatment models differ in the extent to which alliance building aspects of treatment are specified. Some treatment models explicitly describe alliance building stages of treatment (ABFT, Feder and Diamond 2016) or therapeutic practices to build multiple alliances (FFT, Sexton and Alexander, 2004; MDFT, Liddle 2002). For other treatment models, such as family-based CBT (Freeman et al. 2003), no specific alliance building stages or techniques are described. For the latter, the correlation between alliance and outcome might be smaller than for treatment models with a strong emphasis on alliance building practices.

Also, referral to treatment was shown to have a moderating effect in a meta-analytic review on alliance in youth psychotherapy in a way that correlations between alliance and outcome were found to be stronger for help-seeking youth than for youth receiving mandated treatment (McLeod 2011). Another moderating treatment aspect might be the setting in which treatment is conducted. When treatment is (partially) home-based, the therapist enters the home environment of the family. Effectiveness of the treatment might therefore be more dependent on the degree to which the family feels at ease with and trusts the therapist.

Furthermore, sample characteristics can moderate the association between alliance and outcome. In three meta-analytic reviews on alliance in youth psychotherapy, it has been shown that the nature of patients’ problems was a moderating factor: In two reviews, alliance correlated more strongly with outcome for youth with externalizing problem behavior than for youth with internalizing problems (McLeod 2011; Shirk and Karver 2003). A third review indicated that for youth dealing with substance abuse and mixed problems alliance correlated more strongly with treatment outcome than for youth dealing with eating disorders (Shirk et al. 2011). In two of these meta-analytic reviews, age of youth also proved to have a moderating effect, with stronger correlations between alliance and outcome for younger children compared to adolescents (McLeod 2011; Shirk and Karver 2003). Another moderating sample characteristic is shown in a study on alliance and outcome in home-based family therapy, where a stronger correlation between alliance and outcome was found for fathers than for mothers (Johnson et al. 2002). This suggests that gender can moderate the effect of alliance on outcome.

Lastly, it can be reasoned that cultural differences play a role in how important the alliance is in enhancing favorable treatment outcomes, especially in family-involved treatment. For example, in more collectivist cultures the within-family alliance or the extent to which alliances with multiple family members are unbalanced might be of more influence on treatment outcome compared to more individualist cultures. This is illustrated in a study on ethnic background, therapeutic alliance and retention in functional family therapy (FFT), in which unbalanced alliances between family members predicted treatment dropout for Hispanic American families, but not for Anglo-American families (Flicker et al. 2008).

### Present Study

To date, no meta-analytic review of alliance and outcome in family-involved treatment for youth problems has been published that also focused on moderators of the association between alliance and outcome and included studies published since 2008. The present study meta-analytically summarizes research findings on alliance and treatment outcome in family-involved treatment for youth problems over the past three decades. The purpose is to provide accurate estimates of the associations between the level of alliance and treatment outcome, alliance change scores and treatment outcome, and split alliances and treatment outcome, paying particular attention to both within- and between-study variabilities by performing moderator analyses in a multilevel meta-analysis. The analyses therefore ensure maximum use of the available data and provide valuable insight into the process of building, maintaining and measuring alliance in order to enhance positive outcome in family-involved treatment for youth problems.

## Methods

### Sample of Studies

To obtain studies for this article, we conducted the search as prescribed by PRISMA (Moher et al. 2009). Nine databases relevant to the field of this study were searched: Wiley Online Library, Eric, Academic Search Premier, PubMed, Medline, PsycInfo, PsycBooks, Web of Science and ProQuest. The following combination of search terms was used for titles, abstracts and keywords: (“alliance” OR “bond”) AND (“youth” OR “child*” OR “adolescent*” OR “teen*” OR “parent*”) AND (“famil*” OR “system*” OR “multisystem*”) AND (“outcome” OR “effect*” OR “efficacy” OR “dropout” OR “retention”). In addition, retrieved articles were cross-referenced, and Google Scholar was hand searched. Scholars with an expertise on alliance in family therapy were asked if they had any unpublished data of interest for this study. If studies were not retrievable from databases, authors were contacted. Four unpublished dissertations could not be included, because we could not trace the authors or the authors did not respond to our request. The search was completed in October 2017.

**Fig. 1:**
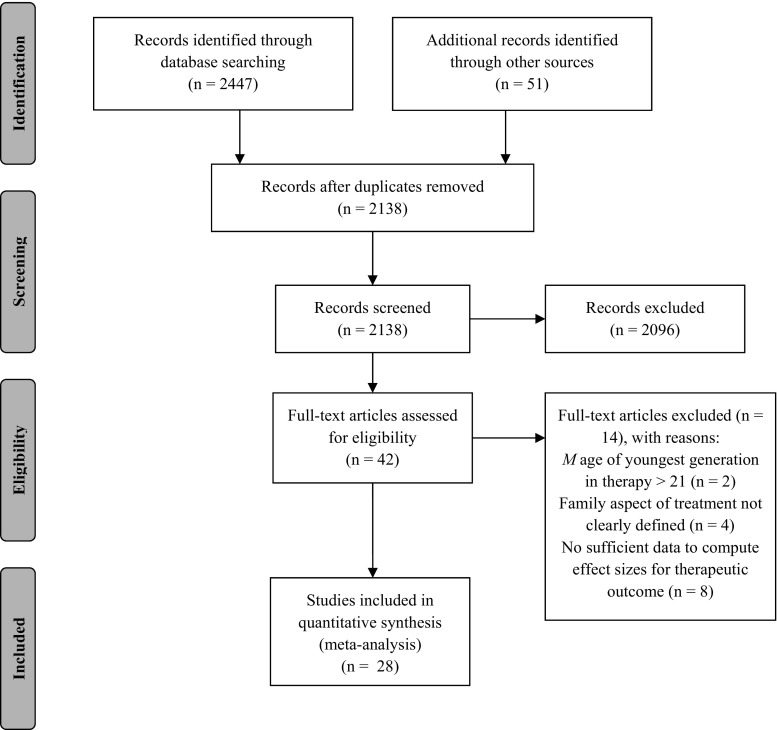
Flow diagram for the search and identification of studies

Studies were included in the meta-analysis if: (a) treatment was conducted for youth problems or for youth being at risk as a result of parental or family problems, (b) treatment was family-involved: In addition to the targeted youth, at least one other family member was actively involved in multiple therapy sessions, resulting in multiple interdependent alliances during treatment, (c) targeted youth had an average age under 21, (d) one or more measures of alliance, working alliance, therapeutic alliance or another measure regarding the emotional bond between client and therapist, agreement or collaboration on goals or tasks between client and therapist, within-family alliance or family therapist alliance were included, (e) one or more measures of treatment outcome on youth, parent or family functioning or retention measured during, at the end or at follow-up of treatment were included, (f) a correlation between the measures mentioned in criteria (d) and (e) was examined regardless of study design, (g) the study report was available in full text and (h) the study report was written in English, Dutch or German. A flow diagram of the search strategy and screening process is depicted in Fig. 1.

We included 28 studies (*k* = 23 published studies, *k* = 4 unpublished dissertations, *k* = 1 unpublished paper), reporting on 21 independent samples comprising a total of *N* = 2126 families. An overview of included studies and their characteristics is shown in Table 1. An overview of sample characteristics for each study is shown in Table 2.

**Table 1:** Summary of studies included in the meta-analysis

Study	Study quality^b^	Treatment model	Treatment setting	Type of TA	TA measure	TA timing	TA Rater	Outcome domain	Outcome timing	Outcome rater	*N* families	*N* individuals^d^	*N* ES^e^
Bachler et al. (2016)	30	TAF	HB	P/Y^c^	CP-TAF	Imp	T	GT, PF, YS	EOT	P	304	n.r.	8
Bennun (1989)	6	FT	C	P	TS	E	P	GT, YS + PF	EOT	P	35	26	5
				Y	TS	E	Y	YS + PF	EOT	P	17	17	
Chinchilla (2007)^a1^	27	MDFT	n.r.	Y	VTAS-R	E	O	R, YS	EOT, FU	Y, P, OM	68	66	11
Dauber (2004)^a1^	25	MDFT	n.r.	Y	VTAS-R	E	O	YS	EOT, FU	Y	63	61	6
Escudero et al. (2008)^f^	24	FT	C	Y + P	SOFTA-O	E, M	O	GT	DT	Y, P	37	82	16
				F	SOFTA-O	E, M	O	GT	DT	Y, P		82	
Feder and Diamond (2016)	20	ABFT	C	P	VTAS-R	M	O	YS	EOT	Y	19	19	2
Flicker et al. (2008)^f^	22	FFT	C	Y	VTAS-R	E	O	R	EOT	T	86	43	6
				P	VTAS-R	E	O	R	EOT	T		43	
				S	VTAS-R	E	O	R	EOT	T		43	
Forsberg et al. (2014)^2^	18	FBT	C	P	WAI-O	E	O	YS	EOT	OM	38	61	3
				S	WAI-O	E	O	YS	EOT	OM		99	
Forsberg (2011)^a2^	15	FBT	C	Y + P	WAI-O	E	O	YS	EOT	Y	38	99	1
Friedlander et al. (2008)^f3^	19	FT	C	F	SOFTA-O	E	O	GT	DT	P	27	n.r.	2
Friedlander et al. (2012)^3^	17	FT	C	Y	SOFTA-S	E, M	Y	GT	DT	Y, P	20	20	4
				P	SOFTA-S	E, M	P	GT	DT	Y, P		36	
Glueckauf et al. (2002)	22	IFCM	n.r.	Y	WAI-S	E + M (A)	Y	YS, GT	EOT	Y	19	19	20
				P	WAI-S	E + M (A)	P	YS, GT	EOT	P		19	
Hawley and Weisz (2005)	29	CB MH	C	Y	TASC	L	Y	R, YS	EOT	OM, T, Y, P	65	65	10
				P	TASC	L	P	R, YS	EOT	OM, T, Y, P		65	
Hogue et al. (2006)^f1^	28	MDFT	n.r.	Y	VTAS-R	E	O	YS	EOT, FU	Y, P	44	44	20
				P	VTAS-R	E	O	YS	EOT, FU	Y, P		n.r.	
Isserlin and Couturier (2012)	18	FBT	C	Y	SOFTA-O	E, M, L	O	YS, R	EOT	OM, Y, P	14	14	75
				P	SOFTA-O	E, M, L	O	YS, R	EOT	OM, Y, P		n.r.	
				F	SOFTA-O	E, M, L	O	YS, R	EOT	OM, Y, P		n.r.	
Johnson et al. (2006)^f4^	27	HB FT	HB	Y + P	FTAS	L	Y, P	YS	EOT	Y + P	225	456	6
Johnson et al. (2002)^f4^	27	HB FT	HB	Y	FTAS	L	Y	YS, PF	EOT	Y	43	16	18
				P	FTAS	L	P	YS, PF	EOT	P		45	
Keeley et al. (2011)	27	FB CBT	C	Y	TASC	E, M	C, T	YS	EOT	Y, P	23	22	16
				P	WAI	E, M	P, T	YS	EOT	Y, P		22	
Kim (2007)^a^	11	SFBT	C	Y	RRS	E	Y	YS	EOT	Y	25	21	20
				P	RRS	E	P	PF	EOT	P		22	
				Y + P	RRS	E	Y, P	PF	EOT	Y, P		n.r.	
				S	RRS	E	Y, P	PF	EOT	Y, P		n.r.	
Lange (in prep.)	29	MST	HB	P	TAM-R	Imp, E, M, L	P	YS	EOT, FU	P	848	774	18
Pereira et al. (2006)^f^	17	FBT	C	Y	WAI-O	E, L	O	R, YS	EOT, DT	OM	41	36	18
				P	WAI-O	E, L	O	R, YS	EOT, DT	OM		31	
Rienecke et al. (2016)	23	FBT PHP	C, H	Y	WAI-S	E, L	Y	R, YS	EOT	O	56	56	17
				P	WAI-S	E, L	P	R, YS	EOT	O		40	
Robbins et al. (2006)^f1^	20	MDFT	n.r.	Y + P	VTAS-R	E	O	R	EOT	OM	30	n.r.	1
Robbins et al. (2008)^f^	23	BSFT	n.r.	Y	VTAS-R	E	O	R	EOT	OM	31	23	14
				P	VTAS-R	E	O	R	EOT	OM		23	
				Y + P	VTAS-R	E	O	R	EOT	OM		n.r.	
				S	VTAS-R	E	O	R	EOT	OM		n.r.	
Robbins et al. (2003)^f^	16	FFT	n.r.	Y	VTAS-R	E	O	R	EOT	OM	34	29	13
				P	VTAS-R	E	O	R	EOT	OM		29	
				Y + P	VTAS-R	E	O	R	EOT	OM		n.r.	
				S	VTAS-R	E	O	R	EOT	OM		n.r.	
Shelef and Diamond (2008)^f5^	26	MDFT	n.r.	Y	VTAS-R(SF)	E, M, L	O	R, YS	EOT	OM	86	45	4
				P	VTAS-R(SF)	E, M, L	O	R	EOT	OM		34	
				Y + P	VTAS-R(SF)	E, M, L	O	R	EOT	OM, Y		68	
Shelef et al. (2005)^f5^	27	MDFT	n.r.	Y	WAI, VTAS-R	E	Y, O	YS, R	EOT, FT	Y, OM	91	59	23
				P	VTAS-R	E	O	R	EOT	OM		65	
				Y + P	VTAS-R	E	O	R	EOT, FU	OM		110	
Zaitsoff et al. (2008)	20	FBT	C	Y	HRQ	ML	Y	YS	EOT	Y	40	40	4

*TAF* therapeutische ambulante familienbetreung; *FT* family therapy, no specific model; *MDFT* multidimensional family therapy; *ABFT* attachment-based family therapy; *FFT* functional family therapy; *FBT* family-based treatment; *IFCM* issue-specific single-family counseling; *CB MH* community-based mental health; *HB FT* home-based family therapy; *FB CBT* family-based cognitive behavioral therapy; *SFBT* solution-focused brief therapy; *FBT PHP* family-based therapy partial hospitalization program; *BSFT* brief strategic family therapy; *C* clinic; *HB* home-based; *H* hospital; *TA* therapeutic alliance; *Y* youth; *P* parent; *Y* *+* *P*  averaged or added scores of youth and parent; *F* within family; *S* Split: difference scores (youth and parent scores subtracted); *T* therapist; *O* observer; *CP-TAF* compliance collaboration scale for Therapeutische Ambulante Familienbetreung; *TS* therapist scale; *VTAS(-R)* Vanderbilt therapeutic alliance scale (–Revised); *SOFTA (-O/S)* system for observing family therapy alliances (-observer/self-report); *WAI(-S/O)* working alliance inventory (short form/observer version); *TASC* therapeutic alliance scale for children; *FTAS* family therapy alliance scale; *HAQ* helping alliance questionnaire; *TAS* therapeutic alliance scale; *RRS* relationship rating scale; *HRQ* helping relationship questionnaire; *E* early treatment; *M* midtreatment; *L* late treatment; *A* multiple moments averaged or added; *Imp* improvement (alliance change scores); *YS* youth symptom severity or functioning; *PF* parental or family functioning; *R* retention; *GT* goal attainment or therapeutic progress; *EOT* end of treatment; *FU* follow-up; *DT* during treatment; *OM* objectified measure; n. r. not reported

^a^Indicates doctoral dissertation

^b^Study quality reflects the study quality checklist score

^c^Alliance was measured for the primary participant of the therapy, which could be either parent or adolescent

^d^Sample sizes are based on a mean of all available reported analyses

^e^Number of computed effect sizes per study

^f^Study was included in previous meta-analysis on alliance in family therapy (Friedlander et al. 2011)

^1,2,3,4,5^Studies reported on the same or overlapping samples

**Table 2:** Sample characteristics of studies included in the meta-analysis

Study	*N* families	Problem type	Referral to treatment	*Mean* age youth	% male youth	% male adult	% non-caucasian	% non-caucasian therapists
Bachler et al. (2016)	304	Multiproblem families	M	14.6	49	35	n.r.	n.r.
Bennun (1989)	35	Mixed	HS	n.r.	n.r.	n.r.	n.r.	n.r.
Chinchilla (2007)^1^	68	Substance abuse	Mx	15.3	80	n.r.	83	50
Dauber (2004)^1^	63	Substance abuse	Mx	15.3	79	n.r.	81	60
Escudero et al. (2008)	37	Mixed	HS	15.0	40	36	0	0
Feder and Diamond (2016)	19	Internalizing problems	HS	15.5	5	16	74	0
Flicker et al. (2008)	86	Substance abuse	Mx	15.7	84	n.r.	50	33
Forsberg et al. (2014)^2^	38	Eating disorders	HS	14.0	13	41	24	n.r.
Forsberg (2011)^2^	39	Eating disorders	HS	14.0	13	41	24	n.r.
Friedlander et al. (2008)^3^	27	Mixed	R	10.2	n.r.	33	7	10
Friedlander et al. (2012)^3^	20	Mixed	R	13.2	33	41	10	11
Glueckauf et al. (2002)	19	Epilepsy with behavioral problems	R	13.9	53	41	11	n.r.
Hawley and Weisz (2005)	65	Mixed	HS	11.9	59	11	63	n.r.
Hogue et al. (2006)^1^	44	Substance abuse	Mx	15.47	81	n.r.	80	60
Isserlin and Couturier (2012)	14	Eating disorders	HS	14.0	0	n.r.	n.r.	n.r.
Johnson et al. (2006)^4^	225	Multiproblem families	M	14.4	n.r.	36	15	n.r.
Johnson et al. (2002)^4^	43	Multiproblem families	M	14.0	n.r.	27	19	n.r.
Kim (2007)	25	Mixed	HS	13.1	48	19	4	n.r.
Lange (in prep.)	848	Externalizing problems	M	15.3	66	17	n.r.	n.r.
Pereira et al. (2006)	41	Eating disorders	R	15.1	9	n.r.	26	n.r.
Rienecke et al. (2016)	56	Eating disorders	HS	15.8	7	37	7	0
Robbins et al. (2006)^1^	30	Substance abuse	Mx	14.9	80	n.r.	83	20
Robbins et al. (2008)	31	Substance abuse	n.r.	15.7	71	43	100	n.r.
Robbins et al. (2003)	34	Substance abuse	Mx	15.0	59	n.r.	n.r.	n.r.
Shelef and Diamond (2008)^5^	86	Substance abuse	Mx	16.0	73	n.r.	51	33
Shelef et al. (2005)^5^	91	Substance abuse	Mx	16.0	85	n.r.	53	33
Zaitsoff et al. (2008)	40	Eating disorders	n.r.	16.1	3	n.r.	36	n.r.

*HS* help-seeking; *R* recruited (for study); *M* mandated; *Mx* mixed; n.r. not reported

^1,2,3,4,5^Studies reported on the same or overlapping samples

### Coding of Studies

In order to code effect sizes and moderating variables of included studies, we developed a coding form, following guidelines as described by Lipsey and Wilson (2001). All study, sample and methodological features shown in Tables 1 and 2 were coded for moderator analyses. If information on certain moderating variables was missing in the study report, authors were contacted to retrieve additional information. All studies that met inclusion criteria were coded by the first author. For 39% (*k* = 11) of the 28 studies, effect sizes and all included moderator variables other than study quality (see the next paragraph) were independently coded by the second author in order to assess interrater reliability. The intraclass correlation coefficient (ICC) for double coded effect sizes (*n* = 127) was .82, average ICC for continuous moderator variables was .95 and average Cohen’s kappa for categorical moderator variables was .70. Differences in scores for effect sizes were discussed until agreement was reached.

Study quality was coded and assessed using a study quality checklist (SQC) developed by the third and first author of this article based on the Quality Assessment Tools for Quantitative Studies (QATQS; Thomas et al. 2004), the Quality Index (QI; Downs and Black 1998) and the Cochrane Collaboration’s tool for assessing risk of bias (Higgins et al. 2011). The SQC allows the rating of 15 criteria per study on publication status, selection bias, pretest differences, missing data, reliability and validity of process measures, reliability and validity of outcome measures, attrition, study dropouts and report on treatment and sample size characteristics. Total SQC scores ranged from 6 to 30 on a 0 (low) to 45 (high) scale. In order to assess interrater reliability of the SQC, 22 out of 28 included studies were independently coded by the first author and a master’s graduate student in Forensic Child and Youth Care Sciences. The ICC was .95.

### Calculation of Effect Sizes and Statistical Analyses

For each study, Pearson’s *r* was calculated to estimate the correlation between alliance and outcome. In cases where two treatments were compared with one of them being a family-involved treatment, Pearson’s *r* was calculated only for the sample that received family-involved treatment. Most effect sizes were calculated based on reported standardized regression coefficients, Pearson’s *r* correlations, and means and standard deviations for treatment completers and dropouts. All calculations were based on formulas of Borenstein et al. (2009), Lipsey and Wilson (2001), Rosenthal (1991, 1994) and Rosenthal and DiMatteo (2001). If effect sizes could not be calculated based on the information in the study report, authors were contacted to retrieve additional information. In seven studies, the study reported non-significant correlations, but did not provide sufficient data to calculate an effect size. In these cases, the value of zero was assigned (*n* = 47 effect sizes), which is considered a conservative estimate of the true effect size (Rosenthal 1995). Furthermore, effect sizes were coded as positive if correlations were in the expected direction (i.e., higher levels of alliance, alliance improvement or lower levels of unbalanced alliance were related to more positive therapy outcome), whereas correlations not in the expected direction were coded as negative. In total, 361 effect sizes were computed. Effect sizes on alliance change scores and outcome (*n* = 15, *k* = 3 studies) and on split alliance and outcome (*n* = 17 from *k* = 5 studies) were each analyzed in separate meta-analyses because of the different nature of the alliance.

To prevent extreme effect sizes or moderating variables from having a disproportionate influence on the statistical analyses, we searched effect sizes and continuous moderators for outliers (standardized scores higher than 3.29 or below − 3.29; Assink and Wibbelink 2016). No outliers were found.

Next, each correlation was transformed to Fisher’s Z before combined effect sizes were calculated and moderator analyses were conducted (Assink and Wibbelink 2016) and transformed back into Pearson’s *r* after analyses for ease of interpretation. Effect sizes were interpreted following Cohen’s (1988) guidelines: The effect is considered small if *r* is at least .10, medium if *r* is at least .30 and large if *r* is at least .50.

Most included studies report on multiple informants of alliance, multiple times of measurement and multiple outcomes. Therefore, for most studies more than one effect size was calculated. Traditional meta-analytic approaches are based on the principle that the included subject samples are independent, and thus, including multiple effect sizes based on the same sample violates this principle (Lipsey and Wilson 2001). However, following other recent meta-analyses (e.g., Assink et al. 2015; Van der Stouwe et al. 2014), a multilevel random effects model was used for the calculation of combined effect sizes and for the moderator analyses in order to account for dependency of effect sizes. This approach has been shown as superior to the fixed effects approaches employed in traditional meta-analysis for models with moderators (Van den Noortgate and Onghena 2003).

In the present study, a three-level meta-analytic model was used for analysis of the data, modeling three sources of variance: sampling variance of the observed effect sizes (level 1), variance between effect sizes from the same study (level 2) and variance between studies (level 3). This model was used to calculate an overall estimate of the association between level of alliance and therapeutic outcome, the association between alliance change scores and outcome and the association between split alliances and outcome in family therapy. Furthermore, it was used to obtain estimates of effect sizes by including moderator variables in the model to determine whether the observed variation was explained by study, sample or methodological characteristics of studies.

To perform the statistical analyses using a three-level model, we followed guidelines as described by Assink and Wibbelink (2016). We used the function “rma.mv” of the metafor package in the R environment (version 3.3.1; R Core Team 2016). The R syntax and protocol was written so that during the analyses three sources of variance were modeled. We used the *t*-distribution for testing individual regression coefficients of the meta-analytic models and for calculating the corresponding confidence intervals.

To determine whether moderator analyses should be conducted, we applied the 75% rule of Hunter and Schmidt (1990). They state that when less than 75% of the total variance can be attributed to random sampling error (level 1), heterogeneity at levels 2 (within studies) and 3 (between studies) can be considered substantial, and moderator analyses should be conducted. Because of the small number of studies and effect sizes included in our meta-analyses on split alliance–outcome and alliance improvement–outcome, the more traditional approach of log-likelihood ratio tests might not lead to significant results when in reality there is substantial variance. Applying the 75% rule of Hunter and Schmidt is an appropriate solution to this power problem (Assink and Wibbelink 2016). For the sake of completeness, we also report results of two separate one-tailed log-likelihood ratio tests in which the deviance of the full model was compared with the deviance of a model excluding one of the variance parameters. The sampling variance of observed effect sizes (level 1) was estimated by using the formula of Cheung (2014), as is appropriate for multilevel analysis (Assink and Wibbelink 2016). The log-likelihood ratio tests were one-tailed, whereas all other tests were two-tailed.

When models were extended with categorical moderators consisting of three or more categories, the omnibus test of the null hypothesis that all group mean effect sizes are equal followed an *F*-distribution. We estimated all model parameters using the restricted maximum likelihood estimation method, and before we conducted the moderator analyses, each continuous variable was centered around its mean. To enable analysis of categorical variables with three or more categories, we created (dichotomous) dummy variables (Tabachnick and Fidell 2012). These dummies contain all information included in the original categorical variable. Given that our moderators were tested in multilevel regression analyses, the intercept is the reference category, while the dummies (the number of categories minus one) reveal if, and to what extent, the other categories deviate from the reference category.

### Analysis of Publication Bias

A problem in the overall estimates of effect sizes in a meta-analysis is that studies with non-significant or negative results are less likely to be accepted for publication by journals. Rosenthal (1995) referred to this problem as the “file drawer problem.” Although obtaining and including unpublished studies as best as possible should resolve this problem, we examined file drawer bias by applying two conventional methods. First, we performed Egger regression (Egger et al. 1997), which tests the degree of funnel plot asymmetry as measured by the intercept from regression of standard normal deviates (effect size divided by its standard error) against the estimate’s precision (the inverse of the standard error). A significant Egger regression test is an indicator of funnel plot asymmetry. We performed the funnel plot asymmetry test using the “regtest” function of the metafor package in R (Viechtbauer 2015). To account for the dependency of effect sizes, we added the standard error of the effect size as a moderator to the Egger regression model.

In addition, we performed a trim-and-fill procedure, as described by Duval and Tweedie (2000), to test for indications of overestimation or underestimation of the true overall effect size. By using the trim-and-fill procedure, a funnel plot can be drawn, showing whether studies or effect sizes are missing on the left or right side of the distribution of effect sizes. A funnel plot with missing effect sizes on the left side of the distribution is an indication that the overall estimate is an overestimation of the true effect. When the funnel plot indicates missing effect sizes on the right side of the distribution, it is expected that the overall effect size is an underestimation of the true effect. These trim-and-fill analyses were performed for all associations using all available effect sizes in R with the function “trimfill” of the metafor package (Viechtbauer 2015).

## Results

### Correlation Between Alliance and Outcomes

Table 3 shows the overall effect sizes for the meta-analyses on level of alliance and outcome, split alliances and outcome and alliance change scores and outcome. The effect size for the relation between level of alliance and outcome was significant (*r* = .183; 95% CI .100, .265; *p* < .001), indicating that higher levels of therapeutic alliance are related to better outcomes of family-involved treatment. The estimate was calculated from data of 20 independent samples reporting on 329 effect sizes. The effect size for the correlation between split alliance and outcome was not significant (*r* = .106; CI − .124, .327; *p* = .343). This estimate was calculated from 5 study samples reporting on 17 effect sizes. The effect size for the correlation between alliance change scores and outcome just failed to reach significance, showing a trend (*r* = .281, CI − .023, .538; *p* = .067), which suggests that alliances that improve during the treatment process might lead to more favorable treatment outcomes. This estimate was calculated from 3 study samples reporting on 15 effect sizes.

**Table 3:** Results for the overall mean effect sizes based on three-level mixed effects models

Type of effect size *r*	# studies^*a*^	# ES	Mean *r* (SE)	95% CI	Sig. mean *r*	% var. level 1	σ^2^ level 2	% var. level 2	σ^2^ level 3	% Var. level 3
Level of alliance–outcome^1^	20	329	.183 (.044)	.100, .265	< .001***	19.6	.044***	48.2	.029***	32.2
Split alliance–outcome^2^	5	17	.106 (.109)	− .124, .327	.343	42.2	.015	15.3	.042*	42.5
Alliance change score–outcome^3^	3	15	.281 (.145)	− .023, .538	.067	5.2	.004	6.4	.058***	88.3

*ES* effect size; *CI* confidence interval; σ^2^ level 2 variance between effect sizes (within studies); σ^2^ level 3 variance between effect sizes (between studies)

^*a*^The number of studies reflects the number of independent samples

*p < .05, **p < .01, ***p < .001

Results of Egger analysis: ^1^ *t* = 12.58, *p* < .001; ^2^ *t* = − .48, *p* = .64; ^3^ *t* = 6.85, *p* < .001

### Moderator Analyses

When applying the 75% rule of Hunter and Schmidt (1990), we concluded that for all three meta-analyses less than 75% of the total variance could be attributed to random sampling error (level 1), and heterogeneity at levels 2 and 3 could be considered substantial. We therefor conducted moderator analyses for all three meta-analyses.

### Moderator Analyses on Level of Alliance and Outcome Correlation

The results of the moderator analyses on the level of alliance and outcome correlation are depicted in Table 4.

**Table 4:** Results of moderator analyses based on three-level mixed effects models for level of alliance and treatment outcome

Moderator	# studies^*a*^	# ES	Mean *r* (SE)	95% CI	β (95% CI)	Test statistic	*p*	σ^2^ level 2	σ^2^ level 3
Study quality	20	329	.421 (.160)**	.130, .645	− .012 (− .027, .002)	*F* (1, 327) = 2.872	.091	.043***	.031***
Sample characteristics									
Problem type general	20	329				*F* (1, 327) = 2.031	.155	.044***	.028***
Youth problems	16	278	.154 (.048)**	.060, .244					
Mixed youth parent/family problems	4	51	.299 (.096)**	.117,.461	.152 (− .058, .350)				
Problem type	20	329				*F* (5, 323) = 1.139	.339	.044***	.027***
Drug abuse youth	3	77	.112 (.093)	− .070, .287					
Eating disorders youth	4	116	.206 (.086)*	.040, .361	.097 (− .151, .332)				
Internalizing problems youth	1	14	.444 (.156)**	.167, .656	.350 (− .007, .619)*				
Externalizing problems youth	1	15	− .004 (.172)	− .347, .326	− .116 (− .466, .265)				
Multiproblem families	2	34	.118 (.125)	− .128, .352	.007 (− .293, .319)				
Mixed	5	73	.203 (.077)**	.054, .344	.093 (− .143, .319)				
Average age youth	18	313	.794 (.286)***	.465, .930	− .062 (− .101, − .022)**	*F* (1, 311) = 9.435	.002**	.045***	.011***
% Male youth	18	298	.424 (.084)**	.082 (.390)	− .158 (− .431, .140)	*F* (1, 296) = 1.098	.296	.045***	.029***
% Male adult	11	105	.140 (.049)**	.044, .234	.059 (− .094, .211)	*F* (1, 103) = .584	.446	.000	.016***
% Non-caucasian	17	238	.199 (.059)***	.085, .309	− .071 (− .320, .178)	*F* (1, 236) = .315	.575	.047***	.020***
% Non-caucasian therapists	7	110	.193 (.081)*	.035, .341	− .186 (− .671, .299)	*F* (1, 108) = .579	.448	.078***	.013
Referral source	18	317				*F* (3, 313) = 2.937	.033*	.044***	.023***
Recruited for study	3	44	.264 (.100)**	.073, .436					
Help-seeking	9	154	.277 (.061)***	.161, .383	.014 (− .214, .239)				
Mandated	1	24	.116 (.160)	− .198, .411	− .151 (− .527, .219)				
Mixed mandated/help-seeking	5	95	.011 (.075)	− .136, .159	− .253 (− .466, − .012)*				
Treatment characteristics									
Treatment model	20	329				*F* (7, 321) = 1.886	.071	.044**	.019**
MDFT	2	65	.114 (.102)	− .088, .308					
FBT	5	116	.204 (.075)**	.059, .340	.091 (− .159, .330)				
FB CBT	1	12	.523 (.164)***	.256, .720	.435 (.083, 691)*				
FFT	2	15	− .124 (.123)	− .353, .119	− .235 (.506, .078)				
MST	1	15	.183 (.074)*	.040, .319	.070 (− .178, .310)				
Other	5	58	.119 (.160)	− .194, .410	.005 (− .355, .364)	.			
Treatment setting	15	225				*F* (2, 222) = 364.015	.175	.000	.037***
Home-based	2	39	.018 (.136)	− .246, .288					
Outpatient clinic	12	169	.265 (.060)***	.152, .371	.248 (− .045, .498)				
Hospital or residential treatment	1	17	.067 (.194)	− .319, .309	.049 (− .399, .478)				
Alliance characteristics									
Type of alliance	20	329				*F* (3, 325) = .028	.891	.045***	.029***
Youth–therapist	17	116	.195 (.050)***	.100, .288					
Parent–therapist	18	132	.168 (.048)***	.075, .259	− .028, (− .106, .052)				
(Within) family/therapist	4	33	.208 (.081)**	.052, .354	.013 (− .134, .159)				
Youth + parent–therapist (added or averaged)	8	48	.191 (.060)**	.075, .301	− .005 (− .108, .99)				
Alliance rater	20	329				*F* (4, 324) = .649	.628	.044***	.029***
Youth	10	58	.179 (.059)**	.065, .289					
Parent	9	67	.184 (.063)**	.063, .300	.005 (− .105, .115)				
Therapist	1	6	.347 (.165)*	.035, .598	.179 (− .145, .468)				
Observer	11	193	.186 (.056)***	.078, .289	.007 (− .126, .140)				
Youth + parent averaged or added	2	5	.007 (.153)	− .289, .301	− .172 (− .445, .129)				
Development of measure	20	329				*F* (1, 327) = 1.340	.248	.044***	.028***
For individual therapy	16	208	.272 (.092)**	.099, .429					
For family therapy	4	121	.158 (.049)**	.063, .250	− .119 (− .313, .084)				
Alliance construct	20	329				*F (5, 323)* = *.445*	.817	.045***	.026***
Bond	5	24	.154 (.080)	− .004, .303					
Goal	3	15	.182 (.096)	− .006, .355	.028 (− .167, .221)				
Task	3	15	.145 (.096)	− .043, .323	− .009 (− .203, .187)				
Goal and task	2	54	.275 (.106)**	.071, .457	.153 (− .069, .360)				
Bond, goal and task	16	182	.164 (.047)***	.072, .253	.126 (− .109, .350)				
Within-family alliance	3	39	.300 (.099)**	.112, .465	.011 (− .151, .173)				
Alliance timing	20	329				*F* (3, 325) = 997.763	.014*	.041***	.029***
Early treatment	15	204	.153 (.047)**	.062, .242					
Midtreatment	6	35	.205 (.069)**	.072, .330	.053 (− .070, .174)				
Late treatment	7	56	.208 (.067)*	.021, .278	− .001 (− .124, .122)				
Averaged or added	4	34	.326 (.079)***	.228, .496	.230 (.090, .360)**				
Outcome characteristics									
Outcome domain	20	329				*F* (3, 325) = 1.609	.187	.044***	.025***
Youth symptom severity or functioning	15	222	.167 (.045)***	.081, .251					
Parental or family functioning	1	6	.020 (.150)	− .270, .306	− .148 (− .410, .136)				
Retention	9	69	.141 (.055)*	.033, .245	− .027 (− .121, .067)				
Goal attainment, therapeutic progress	4	32	.323 (.089)***	.158, .470	.164 (.-017, .335)				
Outcome rater	20	329				*F* (6, 322) = .890	.502	.044***	.030***
Youth	11	111	.163 (.054)**	.059, .265					
Parent	9	77	.235 (.057)***	.125, .337	.074 (− .026, .171)				
Therapist	3	14	.176 (.103)	− .027, .364	.012 (− .200, .224)				
Observer	2	10	.175 (.112)	− .047, .380	.011 (− .200, .222)				
Objectified measure	7	89	.132 (.062)*	.010, .250	− .032 (− .143, .079)				
Youth and parent combined	4	27	.167 (.084)*	.004, .322	.004 (− .169, .176)				
Youth and data combined	1	1	.567 (.298)*	.039, .847	.445 (− .132, .796)				
Outcome timing	20	329				F (2, 326) = .117	.890	.044***	.031***
End of treatment	18	257	.189 (.046)***	.099, .275					
Follow-up	3	42	.165 (.067)*	.036, .289	− .024 (− .128, .082)				
During treatment	3	30	.168 (.069)	− .019, .344	− .021 (− .209, .168)				

*ES* effect size; *CI* confidence interval; σ^2^ *level* 2 variance between effect sizes (within studies); σ^2^ *level* 3 variance between effect sizes (between studies)

^*a*^The number of studies reflects the number of independent samples

*p < .05, **p < .01, ***p < .001

#### Alliance Characteristics

Alliance timing showed a significant moderating effect, with higher correlations when several moments of measurement were averaged or added than for early, midtreatment or late treatment measurement alone. There were no significant moderator effects for type of alliance, alliance rater (informant), alliance construct or alliance measures specifically developed for family therapy.

#### Treatment Characteristics

Treatment model just failed to reach significance, showing a trend indicating a larger effect for alliance in the context of family-based cognitive behavioral therapy compared to alliance in the context of other treatment models. There were no significant moderating effects for treatment setting.

#### Outcome Characteristics

There were no significant moderating effects for outcome domain, outcome rater or outcome timing.

#### Sample Characteristics

A significant moderating effect was found for referral source, indicating a larger effect for help-seeking clients compared to other populations. Furthermore, a significant moderating effect was found for average age of youth in the sample, indicating that for younger children the correlations between alliance and outcome were higher. There were no significant moderating effects for percentage of male youth, male adults, non-caucasian clients and non-caucasian therapists. Also, there was no significant moderating effect for problem type.

*Study quality* just failed to reach a significant moderating effect.

### Moderator Analyses on Split Alliance and Outcome Correlation

Results of the moderator analyses on the association between split alliance and outcome are depicted in Table 5. Categorical variables with only one category represented in the total sample and continuous variables with data on less than one-third of effect sizes in the total sample were excluded from analyses.

**Table 5:** Results of moderator analyses based on three-level mixed effects models for split alliances and treatment outcome

Moderator	# studies^*a*^	# ES	Mean *r* (SE)	95% CI	β (95% CI)	Test statistic	*p*	σ^2^ level 2	σ^2^ level 3
Study quality	5	17	.839 (.282)*	.217, .897	− .041 (− .074, − .008)*	*F* (1, 15) = 7.122	.018*	.015	.007
Sample characteristics									
Problem type	5	17				*F* (2, 14) = 5.347	.019 *	.017	.002
Drug abuse youth	2	8	− .137 (.093)	− .325, .060					
Eating disorders youth	1	2	.179 (.157)	− .160, .477	.307 (− .076, .611)				
Mixed problem types	2	7	.308 (.105)*	.083, .487	.419 (.146, .633)**				
Average age youth	5	17	.970 (.898)	− .769, 1.000	− .135 (− .335, .076)	*F (*1, 15) = 1.871	.191	.016	.030
% Male youth	5	17	.327 (.274)	− .253, .734	− .395 (− .887, .517)	*F* (1, 15) = .809	.383	.016	.042
% Male adult	3	13	.046 (.175)	− .330, .410	.069 (− .377, .514)	*F* (1,11) = .115	.741	.000	.069*
% Non-caucasian	4	15	− .106 (.215)	− .518, .346	.370 (− .076, .816)	*F* (1,13) = 3.207	.097	.000	.140
Treatment characteristics									
Treatment model	5	17				*F* (2, 14) = 31.544	.849	.016	.078*
FBT	1	2	.177 (.307)	− .464, .696					
FFT	2	4	.179 (.220)	− .291, .580	.002 (− .681, .684)				
Other	2	11	.018 (.211)	− .414, .444	− .159 (− .754, .578)				
Alliance characteristics									
Alliance rater	5	17				*F* (1, 15) = .610	.447	.016	.046
Observer	4	12	.062 (.127)	− .207, .323					
Youth + parent averaged or added	1	5	.274 (.244)	− .245, .671	.216 (− .362, .816)				
Outcome characteristics									
Outcome domain	5	17				*F* (1, 15) = .886	.361	.016	.041
Youth symptom severity or functioning	2	7	.229 (.171)	− .134, .538					
Retention	3	12	.024 (.139)	− .267, .311	.206 (− .593, .258)				
Outcome rater	5	17				*F* (3, 13) = .100	.959	.017	.149*
Youth	1	5	.274 (.385)	− .535, .821					
Therapist	1	2	.022 (.390)	− .869, .723	− .260 (− .907, .758)				
Objectified measure	2	8	.046 (.281)	− .578, .585	− .235 (− .865, .687)				
Youth and objectified measure combined	1	2	.177 (.423)	− .615, .791	− .102 (− .876, .818)				

*ES* effect size; *CI* confidence interval; σ^2^ *level* 2 variance between effect sizes (within studies); σ^2^ *level* 3 variance between effect sizes (between studies)

^*a*^The number of studies reflects the number of independent samples

*p < .05, **p < .01, ***p < .001

A moderating effect was found for study quality, indicating that higher correlations between split alliance and outcome were found within studies with lower study quality. Problem type also showed a significant moderating effect, with higher correlations between split alliance and outcome for populations with mixed problem types compared to populations dealing with drug abuse or eating disorders. No moderating effects were found for other sample characteristics or for treatment, alliance or outcome characteristics.

### Moderator Analyses on Alliance Change Scores and Outcome Correlation

Results of the moderator analyses on the association between alliance change scores and outcome are depicted in Table 6. Categorical variables with data for only one category, continuous variables with data on less than one-third of effect sizes in the total sample and variables with data for only one study were excluded from analyses.

**Table 6:** Results of moderator analyses based on three-level mixed effects models for alliance change scores and treatment outcome

Moderator	# studies^*a*^	# ES	Mean *r* (SE)	95% CI	β (95% CI)	Test statistic	*p*	σ^2^ level 2	σ^2^ level 3
Study quality	3	15	.981 (1.000)	− 1.000, 1.000	− .070 (− .380, .254)	*F* (1, 13) = .211	.653	.004	.100***
Sample characteristics									
Problem type general	3	15				*F* (1, 13) = .084	.777	.004	.113**
Youth problems	1	8	.248 (.240)	− .270, .654					
Mixed youth and parent/family problems	2	7	.357 (.325)	− .355, .801	.121 (.653, .771)				
Problem type	3	15				*F* (2, 12) = 7.681	.007**	.004	.004
Internalizing problems youth	1	4	.471 (.135)**	.212, .668					
Externalizing problems youth	1	3	.020 (.078)	− .150, .188	− .455 (− .682, − .149)**				
Multiproblem families	1	8	.357 (.072)***	.214, 485	− .136 (− .440, .194)				
Average age youth	3	15	.878 (.709)	− .497, .997	− .079 (− .215, .059)	*F* (1, 13) = 1.526	.239	.004	.045**
% Male youth	3	15	.908 (.762)	− .570, .999	− .974 (− 1.000, .923)	*F* (1, 13) = 1.534	.237	.004	.045
% Male adult	2	11	− .812 (.278)**	− .945, − .449	1.000 (− .978, 1.000)***	*F* (1, 9) = 22.858	<.001***	.004	.001
% Non-caucasian	3	15	.374 (.328)	− .305,.801	− .656 (− 4.455, 3.143)	*F* (1,13) = .139	.715	.004	.107***
Referral source	3	15				*F* (2, 12) = 7.681	.007**	.004	.004
Help-seeking	1	4	.471 (.135)**	.212, .668					
Mandated	1	8	.357 (.072)***	.214, 485	− .136 (.440, .194)				
Mixed mandated/help-seeking	1	3	.020 (.078)	− .150, .188	− .455 (− .682, − .149)**				
Treatment characteristics									
Treatment model	3	15				*F* (2, 12) = 7.681	.007**	.004	.004
FB CBT	1	4	.471 (.135)**	.212, 668					
MST	1	3	.020 (.078)	− .150, .188	− .491 (− .833, − .149)**				
Other	1	8	.357 (.072)***	.214, .485	− .136 (− .440, .194)				
Treatment setting	3	15				*F* (1, 13) = .918	.355	.004	.061***
Home-based	2	11	.195 (.175)	− .183, .523					
Outpatient clinic	1	4	.471 (.268)	− .082, .802	.303 (− .374, .769)				
Alliance characteristics									
Type of alliance	3	15				*F* (2, 12) = 1.588	.244	.004	.043
Youth–therapist	1	2	.488 (.232)*	.018, .781					
Parent–therapist	1	5	.151 (.165)	− .208, .515	− .364 (− .700, .105)				
Youth + parent–therapist (averaged or added)	2	8	.357 (.208)	− .086, .682	− .158 (.691, .485)				
Alliance rater	3	15				*F* (2, 12) = 23.918	<.001***	.004	.000
Youth	1	1	.707 (.235)**	.347, .886					
Parent	2	4	.034 (.043)	− .060, .126	− .848 (− .880, − .310)**				
Therapist	2	10	.375 (.031) ***	.291, .414	− .468 (− .775, .016)				
Outcome characteristics									
Outcome domain	3	15				*F* (2, 12) = .750	.493	.004	.069 **
Youth symptom severity or functioning	3	8	.302 (.160)	− .039, 580					
Parental or family functioning	1	4	.212 (.174)	− .167, .536	− .097 (− .324, .142)				
Goal attainment or therapeutic progress	1	3	.277 (.175)	− .100, .598	− .028 (− .265, .213)				
Outcome rater	3	15				*F*(2, 12) = .782	.479	.004	.059**
Youth	1	1	.275 (.201)	− .161, .621					
Parent	2	10	.192 (.171)	− .182, .517	− .088 (− .322, .156)				
Youth and parent combined	1	4	.471 (.264)	− .077, .800	.225 (− .469, .747)				
Outcome timing	3	15				*F* (1, 13) = .308	.588	.006*	.049*
End of treatment	1	2	.301 (.136)*	.004, .535					
Follow-up	3	13	.241 (.154)	− .090, .523	− .055 (− .264, .159)				

*ES* effect size; *CI* confidence interval; σ^2^ *level* 2 variance between effect sizes (within studies); σ^2^ *level* 3 variance between effect sizes (between studies)

^*a*^The number of studies reflects the number of independent samples

*p < .05, **p < .01, ***p < .001

#### Alliance Characteristics

There was a significant moderating effect for alliance rater, with stronger correlations between alliance improvement and outcome for youth informed alliance improvement than for therapist or parent informed alliance improvement. There was no moderating effect for type of alliance.

#### Treatment Characteristics

A significant moderating effect was found for treatment model, with higher correlations between alliance improvement and outcome for family-based CBT compared to MST and other forms of family-involved treatment. There was no moderating effect for treatment setting.

#### Sample Characteristics

Problem type was a significant moderator: Correlations between alliance improvement and outcome were higher for families in treatment for internalizing problems of their children and for multiproblem families compared to families receiving treatment for externalizing problems of their children. Referral source was also a significant moderator, with higher correlations between alliance improvement and outcome for help-seeking or recruited clients than for clients mandated for treatment or populations with mandated as well as help-seeking clients. Furthermore, percentage of male adults within the study sample was a significant moderator, demonstrating higher correlations between alliance change and outcome within samples with a higher percentage of male adults.

There were no significant moderating effects for *outcome characteristics* or for *study quality*.

### Analyses of Publication Bias

**Fig. 2:**
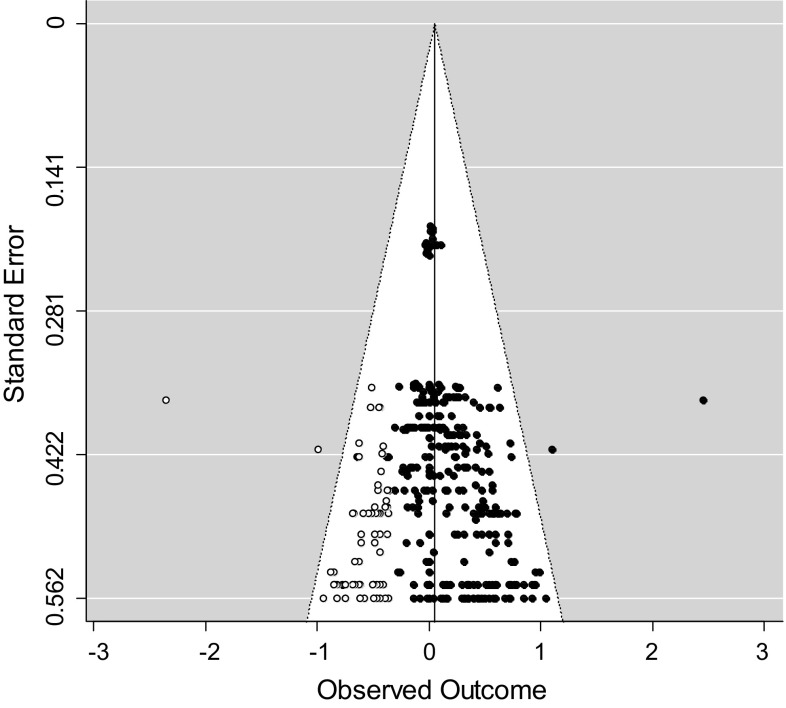
Trim-and-fill plot level of alliance–outcome association

**Fig. 3:**
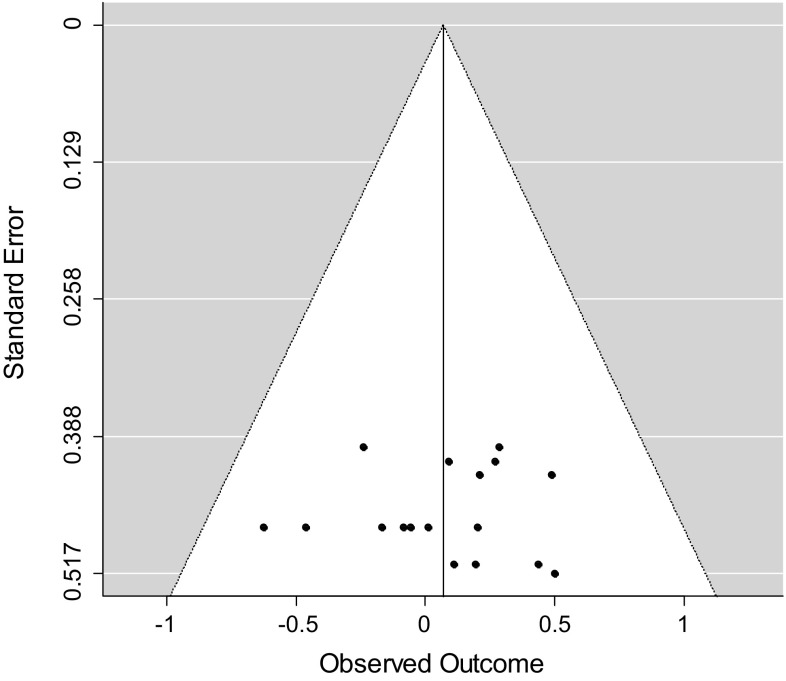
Trim-and-fill plot split alliance–outcome association

**Fig. 4:**
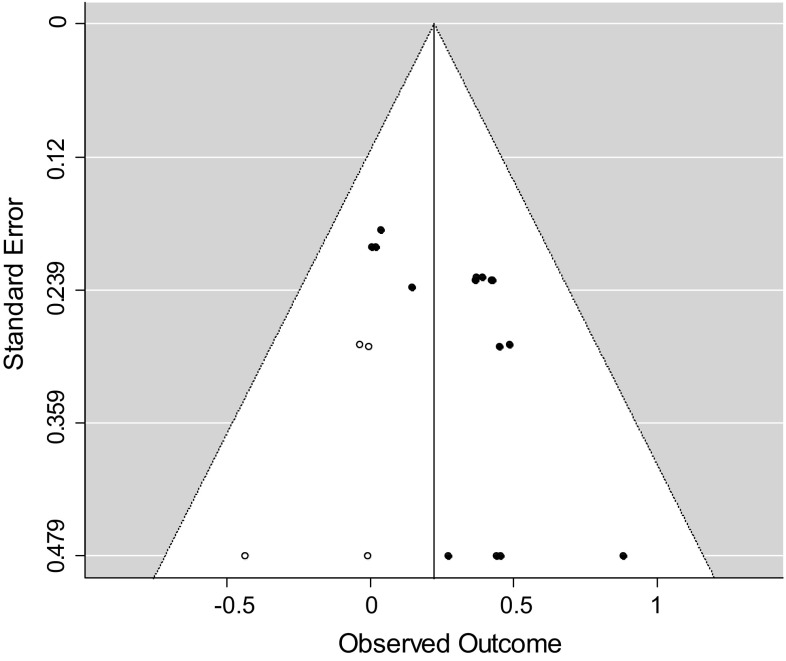
Trim-and-fill plot alliance change score–outcome association

In order to investigate whether publication bias might have distorted the results of our meta-analyses, we applied two methods. Table 3 shows the results of the Egger regression test for each analyzed association. The association between level of alliance and outcome and the association between alliance change scores and outcome showed significant Egger regression tests, indicating funnel plot asymmetry. The funnel plots showing the results of the trim-and-fill procedure are depicted in Figs. 2, 3 and 4. Both trim-and-fill plots for the level of alliance–outcome association and the alliance change scores–outcome association show missing effect sizes on the left side of the distribution, indicating that the overall effect sizes in these meta-analyses may be an overestimation of the true effect. Comparison of confidence intervals revealed that the overall effect size of the level of alliance–outcome association was significantly smaller after trim-and-fill analysis (*r* = .05, *p* < .05) compared to the overall effect size before trim-and-fill analysis. The overall effect size of the correlation between alliance change scores and outcome did not significantly vary from the overall effect size before trim-and-fill analysis.

## Discussion

### The Association Between Alliance and Treatment Outcome

Our findings revealed a significant small to medium correlation between the level of alliance and treatment outcome (*r* = .18), indicating that higher levels of alliance between the therapist and the family lead to more favorable treatment outcomes. This finding is in line with previous meta-analyses on alliance and treatment outcome in youth psychotherapy, showing comparable overall effect sizes, ranging from *r* = .14 to *r* = .22 (Karver et al. 2006; McLeod 2011; Shirk and Karver 2003; Shirk et al. 2011). Meta-analyses on alliance in adult psychotherapy have consistently shown somewhat larger overall effect sizes, ranging from *r* = .21 to *r* = .28 (Horvath and Bedi 2002; Horvath et al. 2011; Martin et al. 2000). Friedlander et al. (2011) performed a meta-analysis on alliance in couple and family therapy, and found an overall effect size of *r* = .26 for both couple and family therapy, and an overall effect size of *r* = .24 for family therapy only.

The fact that the present meta-analysis yielded a somewhat smaller overall effect size for family-involved treatment than the meta-analysis by Friedlander et al. (2011) can be explained by several factors. First, we used stricter inclusion criteria for the family aspect of treatment and included unpublished studies as well as published studies. Second, we used a multilevel model instead of a traditional single-level model as used by Friedlander et al. (2011). It can therefore be expected that the present study provides a more accurate estimate of the overall effect size.

Furthermore, the previous meta-analysis by Friedlander et al. (2011) did not report an analysis of publication bias, which may have led to an overestimation of the true effect size. In the present study, correlations between alliance and treatment outcome reported in studies as non-significant without sufficient data to calculate the true effect size were included, with a conservative estimation of zero. As Rosenthal (1995) pointed out, this conservative estimate of the effect size might lead to an underestimation of the true effect, but simply not using these effect sizes might lead to overestimation of the true effect. To test the hypothesis of underestimation in the present study, we again calculated the overall effect size for the association between the level of alliance and treatment outcome with exclusion of all effect sizes estimated to be zero. The result was a higher overall effect size of *r* = .22 (*p* < .001). However, the Egger and trim-and-fill analyses indicated that the original overall effect size we found for the association between level of alliance and outcome may still be an overestimation of the true effect size due to publication bias.

Contrary to our expectations, we found only a small correlation between split or unbalanced alliances and outcome, which failed to reach significance. This could indicate that for positive treatment outcome it is irrelevant whether the therapist develops balanced therapeutic relationships with all family members or develops a stronger therapeutic relationship with one of the family members compared to other family members. However, when interpreting the results of the meta-analysis on split alliance and treatment outcome, it should be noted that research on split alliances often lacks a clear definition of the central concept as well as a valid and reliable methodology to the concept. Often, raw difference scores are used to investigate the role of split alliances in treatment outcome. Previous research, however, has shown that these difference scores cannot provide valid and reliable tests of informant discrepancy as a predictor (Bartle-Haring et al. 2012; Laird and De Los Reyes 2013).

Results of the analysis on the association between alliance change scores and treatment outcome showed a trend toward significance indicating a moderate association of *r* = .281, which is considerably larger than the correlation between level of alliance with fixed moment measures and treatment outcome (*r* = .18). This might indicate that for the therapist in order to enhance positive treatment outcome, improving alliances with family members during the treatment process might even be more important than developing alliances that remain stable throughout treatment. However, research on alliance change scores related to treatment outcome in family-involved treatment is scarce, and only three studies reporting on alliance change scores could be included in the meta-analysis. This is surprising, given that previous research on alliance in several contexts has shown that alliance can develop in different trajectories during treatment, such as a linear increase in alliance, a fading linear increase in alliance or sudden nonlinear decreases (ruptures) or increases (gains) in alliance (Lange et al. in prep.). How these different developmental trajectories of alliance relate to treatment outcome remains unclear.

### Moderating Variables

The results of our study reveal that the association between alliance and outcome was moderated by several characteristics of alliance measures, treatment and study sample. With regard to alliance measures, we found that the correlation between the level of alliance and outcome was stronger when alliance measures at several time points were averaged or added compared to only early, mid- or late treatment measures of alliance. This finding is in line with a study on the alliance–outcome association in psychotherapy for depressed adults, where the average of alliance score measured at session 3–9 explained 14.7% of the outcome variance, whereas single alliance measures of session 3 explained only 4.7% of the outcome variance (Crits-Christoph et al. 2012). Meta-analytic reviews on the alliance–outcome association in adult and youth psychotherapy have consistently reported stronger correlations between alliance measured during late treatment and alliance measured during early or midtreatment (Horvath et al. 2011; McLeod 2011; Shirk and Karver 2003). However, none of these studies reported on multiple alliance measures averaged or added as a category for timing of alliance measure. If we take into account that our meta-analysis on alliance change scores and treatment outcome showed a marginally significant larger effect size compared to the association between level of alliance and outcome, our findings underline the importance of viewing the alliance as a dynamic process rather than a static, single-moment entity.

No moderating effects were found in any of our analyses for type of alliance informant (youth, parent or observer) or alliance construct. These findings are in line with the findings from the meta-analytic review of Shirk et al. (2011) that did not show moderating effects for any characteristics of the alliance measure. Also, Horvath et al. (2011) found no moderating effect for alliance rater in the association between alliance and outcome in individual adult psychotherapy. However, other meta-analytic reviews have reported larger effect sizes for therapist rated alliance compared to other sources of alliance measurement (Shirk and Karver 2003), or for parent rated alliance compared to other sources of alliance (McLeod 2011). This might indicate that no consistent conclusion can yet be drawn about the role of alliance source in the alliance–outcome association.

Contrary to our expectations, in the association between level of alliance and outcome we found no moderating effect for type of alliance informant (youth, parent or observer), type of alliance (youth–therapist, parent–therapist or family alliance), construct of alliance (bond, goal, task, within-family) or alliance measures designed specifically for family therapy in order to capture systemic aspects of the alliance. Previous studies that found a smaller or less significant effect on treatment outcome for alliance in family-involved treatment compared to individual treatment have underlined the importance of studying alliance in family therapy with instruments that capture systemic aspects of alliance typical of working with multiple family members (Lange et al. in prep.; McLeod 2011). The rationale behind this point of view is that alliance instruments designed for family therapy might lead to a better understanding of the alliance–outcome association in family-involved treatment. However, research on alliance and outcome using specific family therapy alliance measures is still scarce, and in the present study, only four independent samples using a specific family therapy measure of the alliance could be included in the meta-analysis on level of alliance and outcome. Furthermore, out of these four study samples, three samples contained only a small number of families (*n* < 50).

Most of the significant moderating variables were sample characteristics, with different moderators for the three separate meta-analyses. The association between level of alliance and treatment outcome was significantly moderated by average age of youth in the sample, demonstrating stronger correlations when youths were younger. This is in line with findings of McLeod (2011) and Shirk and Karver (2003) showing that in youth psychotherapy associations between alliance and outcome were stronger for younger children compared to adolescents. However, it should be noted that in the present study variance in average age of youth in study samples was small, with the lowest average age of 10.6 and the highest average age of 16.1. Most study samples comprised only families with adolescents, some samples comprised adolescents as well as younger children, and no studies were included with families with children in primary school age only. It is unclear whether our study findings can be generalized to families receiving therapy or treatment due to concerns regarding much younger children. In families with younger children, the role of the child in therapy might not be as active as compared to older youth, resulting perhaps in lower correlations between youth alliance and outcome and higher correlations between parent alliance and outcome.

Another moderating sample characteristic in the association between level of alliance and treatment outcome was referral source, showing stronger correlations between alliance and outcome for clients who were help-seeking or recruited for the study compared to samples with mandated clients or a combination of mandated and help-seeking clients. This finding was replicated in the meta-analysis on alliance change scores and treatment outcome. Two recent studies compared alliance processes in family therapy between voluntary and involuntary clients. These studies revealed that initial between-group differences in the emotional bond with the therapist and the within-family alliance did disappear after four sessions of therapy (Sotero et al. in press; Sotero et al. 2016). Between-group differences in agreement on therapeutic goals and tasks, however, remained after the fourth session. Thus, the difference in the alliance–outcome association between self-referred and involuntary clients might be explained by both timing and dimension of alliance measure. However, no research has yet been published on the relation between specific aspects of alliance processes with mandated clients in relation to treatment outcome. Furthermore, in the present meta-analysis only one study could be included with mandated clients only. Five other included studies reported on samples of both mandated and help-seeking clients, with no reports of specific effect sizes for both groups.

It was surprising that no moderating effects were found for problem type or treatment model in the association between level of alliance and outcome. Several meta-analyses have demonstrated moderating effects for problem type (McLeod 2011; Shirk and Karver 2003; Shirk et al. 2011). In the present study, the sample of included studies was very heterogeneous with regard to problem type and treatment model. As a result, several categories for these variables were represented by only one or two studies. Thus, the fact that the moderating effect of problem type and treatment model failed to reach significance might partly be explained by a lack of statistical power. Nevertheless, this finding underlines the importance of training and supervision for therapists in alliance building techniques in addition to training and supervision of specific treatment model techniques.

In contrast to our expectations, both gender and ethnicity of clients did not moderate the association between level of alliance and outcome. With regard to gender, two previous studies on alliance and outcome in family therapy and couple therapy demonstrated that for male adults other aspects of alliance are important in relation to treatment outcome compared to females (Johnson et al. 2002) and that the correlation between alliance and outcome might be stronger when males have a higher level of alliance with their therapist than their female partners (Symonds and Horvath 2004). With regard to ethnicity, one included study on split alliance and outcome shows a stronger correlation between alliance and outcome for Hispanic families compared to Anglo-American families (Flicker et al. 2008). It should be noted, however, that most of the studies included in the present study made no distinction between alliance–outcome correlations for boys and girls, or father and mothers, or between different ethnical groups. Thus, there was no variance between effect sizes within studies with regard to gender or ethnicity. We did, however, find a significant moderating effect for percentage of male adults in the association between alliance change scores and treatment outcome, demonstrating stronger correlations in samples with less male adults. This might indicate that for fathers, the process of alliance improvement is more predictive of treatment outcome than for mothers.

For outcome measures characteristics, no moderating effects were found in any of the investigated associations in contrast to findings of previous meta-analyses on alliance and outcome in youth or adult psychotherapy (Horvath et al. 2011; McLeod 2011; Shirk and Karver 2003). This indicates that alliance is a significant small predictor of treatment outcome in family-involved treatment, regardless of how and when outcome is measured.

Lastly, there was no moderating effect for study quality in the associations between level of alliance and outcome, and alliance change scores and outcome, although there was a trend toward significance in the first association indicating stronger correlations in studies of less quality. This moderating effect was significant in the association between split alliances and outcome.

### Limitations of the Study

The present study has several limitations. An important methodological limitation is the small number of studies included in the meta-analyses that investigated the association between split alliances and treatment outcome (five studies reporting on 17 effect sizes) and the association between alliance change scores and treatment outcome (three studies reporting on 15 effect sizes). Therefore, conclusions from these analyses should be interpreted with caution and require future re-evaluation when a larger body of evidence has accumulated.

Second, some categorical variables in the moderator analyses contained relatively few studies, which resulted in insufficient statistical power of the analyses. This was the case for all moderator variables in the associations between split alliances and outcome, and alliance change scores and outcome as a result of the small number of studies included in these meta-analyses. For the association between level of alliance and treatment outcome, the problem of insufficient statistical power might especially apply to problem type, treatment setting, treatment model, referral source and several outcome characteristics.

A final limitation is that in the current meta-analysis, alliance–outcome associations were analyzed across a variety of research designs, ranging from uncontrolled pre-post designs to quasi-experimental designs. It could be reasoned that the strength of alliance–outcome associations differs considerably across research designs. Therefore, future research—based on a larger body of evidence than is currently available—may benefit from a fine-grained analysis of the moderating effect of research designs on the alliance–outcome association in general.

### Implications for Future Research

The sample of studies included in the present study shows that the association between alliances processes and treatment outcome has received less attention within specific treatment contexts. Treatment contexts that differing from the regular context of family-involved treatment for youth problems (i.e., family therapy in an outpatient clinic with families seeking help for a specific problem of their adolescent) might lead to different behaviors of clients, demanding different alliance building skills from therapists. Research on alliance in specific contexts, such as home-based interventions, interventions for multiproblem families or families receiving mandated treatment, might lead to a better understanding of how alliance processes are related to outcome within these specific contexts.

Future research on alliance in family-involved treatment could also benefit from investigating the more complex systemic and dynamic aspects of alliance typical of working with families. One of these systemic aspects is the occurrence of split alliances. As pointed out before, the scarce research on split alliances that is available often lacks a clear definition of the concept, and applied methodology in most of these studies might not be appropriate for investigating the role of split alliances in treatment effectiveness. For research on split alliances, applying methods other than using discrepancy scores is recommended, such as multilevel modeling (Bartle-Haring et al. 2012) or polynomial regression (Laird and De Los Reyes 2013).

Furthermore, the use of alliance measures designed specifically for the context of family therapy, such as FTAS (Pinsof et al. 2008) or SOFTA (Friedlander et al. 2006), may help to gain a better understanding of systemic dynamics of alliance in family-involved treatment related to outcome. Although it has been reasoned before that clarifying these systemic dimensions of alliance may help to produce a more accurate estimate of the association between alliance processes and treatment outcome (Friedlander et al. 2006; McLeod 2011), the present study shows that research investigating the within-family or family therapist alliance is still scarce.

Our findings furthermore indicate that research on the role of alliance in family-involved interventions in treatment effectiveness could benefit from viewing the alliance as a dynamic process rather than a static measure at a single time point. However, research on the evolvement of alliance during treatment is still scarce, and questions remain in particular about the relation between specific developmental trajectories of alliance, such as alliance ruptures or sudden alliance gains, and treatment outcome.

Lastly, research on family-involved treatment might benefit from investigating the role of other common factors that have been hypothesized to be important in determining treatment outcome, such as client motivation, expectancies about services and family empowerment (Hoagwood 2005; Karver et al. 2006; Sprenkle and Blow 2004).

## Conclusions

We investigated the association between alliance and treatment outcome in family-involved treatment for youth problems by analyzing data from 28 studies reporting on 21 independent study samples. Our findings demonstrate that a stronger alliance is a small but significant predictor of better treatment outcomes, underlining the importance for therapists to develop strong alliances with family members during treatment. The association between alliance and treatment outcome was stronger when youth in treatment was in their early adolescence compared to late adolescence, when clients in the study sample were help-seeking or recruited for the study instead of mandated for treatment and when alliance measures of several time points during treatment were averaged or added.

Results of our study furthermore indicate that growth of alliance during the treatment process might be a stronger predictor of treatment outcome than alliance measured at a single time point or an average of alliance measures over time. The occurrence of split alliances did not predict treatment outcome. However, only few studies reported on the association between split alliances and outcome and most studies lack a clear definition and appropriate methodology to measure split alliances.

Our study underlines the importance for therapists to build strong individual alliances with all family members involved in treatment as well as to pay attention to systemic aspects of the alliance, such as the within-family alliance, when delivering family-involved treatment for youth problems. Furthermore, our study implicates that training and supervision of (family) therapists should focus not solely on specific treatment model techniques, but also on alliance building techniques in the context of working with multiple family members. Building these multiple alliances remains important throughout the treatment process, regardless of the treatment model. Therapists might enhance treatment outcome by monitoring individual as well as within-family alliances, in order to intervene when alliances are problematic.

Future research should focus on the association between alliance and outcome in specific treatment contexts of family-involved treatment, such as home-based interventions and therapy with involuntary clients. Furthermore, future research could benefit from investigating complex aspects of alliance within family-involved treatment, such as the role of within-family alliance, the occurrence of split alliances and alliance ruptures, to gain fuller understanding of the dynamic role of alliance in family-involved treatment in order to enhance positive treatment outcome.
